# Exposure to quadrivalent influenza vaccine during pregnancy: Results from a global pregnancy registry

**DOI:** 10.1111/irv.12897

**Published:** 2021-09-14

**Authors:** Shaleesa Ledlie, Sonja Gandhi‐Banga, Anju Shrestha, Tamala Mallett Moore, Alena Khromava

**Affiliations:** ^1^ Epidemiology and Benefit Risk Sanofi Pasteur North York Ontario Canada; ^2^ Global Pharmacovigilance Sanofi Pasteur Swiftwater Pennsylvania USA

**Keywords:** Fluzone, IIV4, maternal immunization, passive surveillance, pregnancy registry, quadrivalent influenza vaccine

## Abstract

**Background:**

The Fluzone® Quadrivalent (IIV4, Sanofi Pasteur) Pregnancy Registry was created to monitor vaccine safety during pregnancy (clinicaltrials.gov, NCT01945424). Here, we describe maternal, pregnancy, obstetrical and neonatal outcomes after vaccine exposure in pregnant women between August 2013 and September 2019.

**Methods:**

All women exposed to IIV4 during their pregnancy were eligible for inclusion. Outcomes were prospective (reported following vaccine exposure but before knowledge of pregnancy outcome ascertained through prenatal tests) or retrospective (prenatal tests were undertaken before the exposure was reported).

**Results:**

Among 239 IIV4 vaccine exposure reports received, there were 105 prospective and 10 retrospective reports of maternal adverse events (AEs). The most frequent prospectively reported maternal AEs were medication errors (expired product [*n* = 8, 3.8%]; extra dose [*n* = 7, 3.3%]) and injection site pain (*n* = 7, 3.3%). Among 62 prospectively reported pregnancy and obstetrical events with available follow‐up information, seven AEs were reported, four (6.4%) of which were spontaneous abortions. A further seven AEs were reported among the 29 retrospective pregnancy and obstetrical events with available follow‐up information. Among neonatal outcomes (15 prospective; 28 retrospective), >85% were reported as full‐term births. One premature birth was reported prospectively. Four other neonatal AEs were reported, all retrospectively: two cases of talipes (club foot), one central nervous system anomaly and one atrial septal defect. All infants with available information had normal APGAR scores at 5 minutes.

**Conclusions:**

The frequency of AEs following exposure to IIV4 during pregnancy did not indicate new safety concerns.

## 1 INTRODUCTION

Seasonal influenza causes a substantial disease burden globally, with approximately 3–5 million cases of severe illness and up to 650,000 deaths annually.^1^ Influenza infection during pregnancy, particularly during the second and third trimesters, is associated with an increased risk of developing severe complications such as pneumonia, leading to hospitalization and/or death.^2^, ^3^, ^4^ Women who develop influenza during pregnancy also have an increased risk for adverse obstetric outcomes, including preterm labour and delivery.^2^ Additionally, infants younger than six months old are at an increased risk of influenza‐related mortality and morbidity.^3^, ^5^

Maternal immunization against influenza is thus considered to be an essential component of prenatal care.^6^ Immunization directly protects the mother and indirectly protects their unborn infant through the transfer of transplacental antibodies and secreted IgA antibodies from breastfeeding.^2^, ^7^, ^8^ Infants often respond poorly to vaccines as a result of an immature immune system and are not included in the indication for influenza vaccination, but maternal antibodies may circulate in the infant until six months of age.^7^, ^8^ It is recommended that all pregnant women receive an influenza vaccine irrespective of their trimester of pregnancy.^1^, ^9^, ^10^, ^11^ Nevertheless, vaccine coverage among pregnant women remains low in many countries.^12^, ^13^ In the United States, influenza vaccine coverage for pregnant women increased from 8.8% during the 2002–2003 influenza season to 50.9% for 2011–2012^14^ but continues to remain below the 2020 Healthy People target to vaccinate 80% of pregnant women.^15^ Coverage estimates below 10% have been reported among pregnant women in a number of Southeast Asian countries^16^, ^17^; in a cross‐sectional study in Singapore, fewer than half (46%) of pregnant women knew that influenza vaccination was recommended during pregnancy.^16^ Similarly, during the 2019 flu season in Canada, 45% of pregnant women received the recommended influenza vaccine.^18^

Concerns over vaccine safety have been found to be a major factor contributing to reduced vaccine uptake among pregnant women.^12^, ^19^, ^20^ Safety data collected after vaccine exposure during pregnancy in randomized clinical trials are generally limited to women who are inadvertently vaccinated during pregnancy, as pregnancy tends to be listed as an exclusion criterion at enrolment.^21^ However, numerous observational studies have shown that inactivated influenza vaccination during pregnancy is not associated with an increased risk of pregnancy‐related complications such as preterm birth and spontaneous abortion.^22^, ^23^, ^24^, ^25^

The Fluzone® quadrivalent vaccine (IIV4) is indicated for active immunization against influenza A and B in individuals aged six months or older. IIV4 is currently licensed in 27 countries, including the United States, Canada, Australia and several countries across South America and Asia. The Sanofi Pasteur Fluzone® Quadrivalent Pregnancy Registry (NCT01945424), herein referred to as the IIV4 pregnancy registry, was created to fulfil a US Food and Drug Administration post‐licensure commitment to monitor IIV4 exposure during pregnancy and any corresponding maternal, pregnancy, obstetrical and neonatal outcomes using routine pharmacovigilance surveillance. Here, we describe vaccine exposure and relevant outcomes reported across various countries to the IIV4 pregnancy registry from its initiation in August 2013 to September 2019.

## 2 METHODS

### 2.1 Study design

The IIV4 pregnancy registry was designed in accordance with the 2002 US Food and Drug Administration Guidance for Industry on Establishing Pregnancy Exposure Registries.^26^ The registry conformed with the Health Insurance Portability and Accountability Act (HIPAA) regulations. IIV4 is currently licensed in 27 countries, and pregnancy exposure reports received from any of these countries were eligible for inclusion in the registry. The decision to participate in the registry or to disclose follow‐up information was completely voluntary and participants could withdraw from the study at any time. This study represents routine pharmacovigilance and as such was not subject to Institutional Review Board review and informed consent requirements. Routine pharmacovigilance procedures and follow‐up are exempt from data privacy in the United States according to the Health Insurance Portability and Accountability Act of 1996 (HIPAA), Public Law 104‐191.^27^

### 2.2 Study population

The study population eligible for inclusion in the IIV4 pregnancy registry was all women of reproductive age who were exposed to IIV4 during their pregnancy or within 30 days of their last menstrual period (LMP). The 30‐day window following their LMP ensured that women who may have been exposed immediately preceding conception were monitored; this also helps to account for inaccuracies in participant recall of LMP. For cases where the LMP was not reported, the estimated date of delivery (EDD) was used to approximate the LMP by counting back 280 days.

The exposure status for each report was verified. If not provided in the reporting information, where possible, the gestational week at the time of exposure was calculated by comparing the vaccination date to the LMP.

### 2.3 Pharmacovigilance

All voluntary, spontaneous reports of exposure submitted to the registry by health care practitioners (HCPs) and pregnant women were captured in the Sanofi Global pharmacovigilance database. A recommendation to contact Sanofi Pasteur regarding all exposure occurring during pregnancy for inclusion in the registry is included in the prescribing information for a number of countries including the United States and Canada^28^ and on the Sanofi Pasteur pregnancy registry website.^29^ All individuals reporting pregnancy exposure to the vaccine were sent a Pregnancy Report Form to collect any missing or additional follow‐up information on relevant maternal, pregnancy, obstetrical and neonatal outcomes, including any adverse events (AEs). For pregnancies ending in a live birth, a structured Infant Data Collection Form was sent 6 months after the EDD to collect follow‐up information on the infant's condition and the diagnosis of any congenital anomalies. If either of these forms were not returned, three follow‐up reminders were sent by Sanofi Pasteur. If the initial reporter did not have access to information on the infant, an attempt to obtain the contact information of the infant's paediatrician or doctor was made. The initial pregnancy exposure reports were separated for analysis depending on whether the case was prospective or retrospective in order to reduce reporting bias. Pregnancy exposure reports were classified as prospective if the report was made following vaccine exposure but before knowledge of the pregnancy outcome was ascertained through prenatal testing. A report was considered to be retrospective if prenatal tests had already been undertaken before the exposure was reported. Reports with no information available on the timing of prenatal tests were considered prospective.

All reported AEs were coded according to the Medical Dictionary of Regulatory Activities preferred terms (MedDRA PTs), including diagnoses and symptoms.^30^ Each event was treated independently, such that the total number of events was assessed separately from the number of women for whom AEs were reported.

### 2.4 Outcomes

Exposure reports were categorised according to three predetermined outcome categories: (i) maternal outcomes, (ii) pregnancy and obstetrical outcomes and (iii) neonatal outcomes. Maternal events were defined as those impacting maternal health but independent of the pregnancy (e.g., injection site reactions); medication errors, including vaccine storage and administration issues, were included in line with good pharmacovigilance practices.^31^, ^32^ Pregnancy and obstetrical outcomes were those directly affecting pregnancy, labour or delivery (e.g., spontaneous abortion); neonatal outcomes were those that were directly related to the infant, evaluated at birth or within the first 28 days of life (including congenital abnormalities). For infants reported to have been born full‐term and for whom the information was available, the reported birth weight and APGAR scores at the 5‐minute mark were also reported. All AEs, including those reported through follow‐up information and regardless of outcome category, were evaluated for medical seriousness at the time of receipt. Serious AEs (SAEs) included congenital anomalies, persistent or significant disability, life‐threatening, hospitalizations, death and other medically serious events.

### 2.5 Statistical methods

The IIV4 pregnancy registry is descriptive, and no predetermined target sample size was established. The baseline characteristics of the exposure reports were presented using descriptive statistics. The frequency of AEs within each outcome category and corresponding 95% confidence intervals (CIs) were calculated, stratified by prospective or retrospective reporting. The Agresti–Coull binomial proportions CI was utilized, supplemented by the Jeffreys interval when indicated.^33^ Losses to follow‐up were accounted for by adjusting the denominator used to calculate frequencies within each outcome category. When assessing maternal outcomes, all exposure reports were included in the denominator, regardless of the availability of follow‐up information. For pregnancy and obstetrical outcomes, all cases where at least one successful follow‐up was completed were included. For neonatal outcomes, all cases with follow‐up information on the infant were included. For birth weight and APGAR score, the denominator included infants for whom this information was available. All analyses were performed using SAS Enterprise Guide 7.1 (SAS Institute, Cary NC).

## 3 RESULTS

**FIGURE 1:**
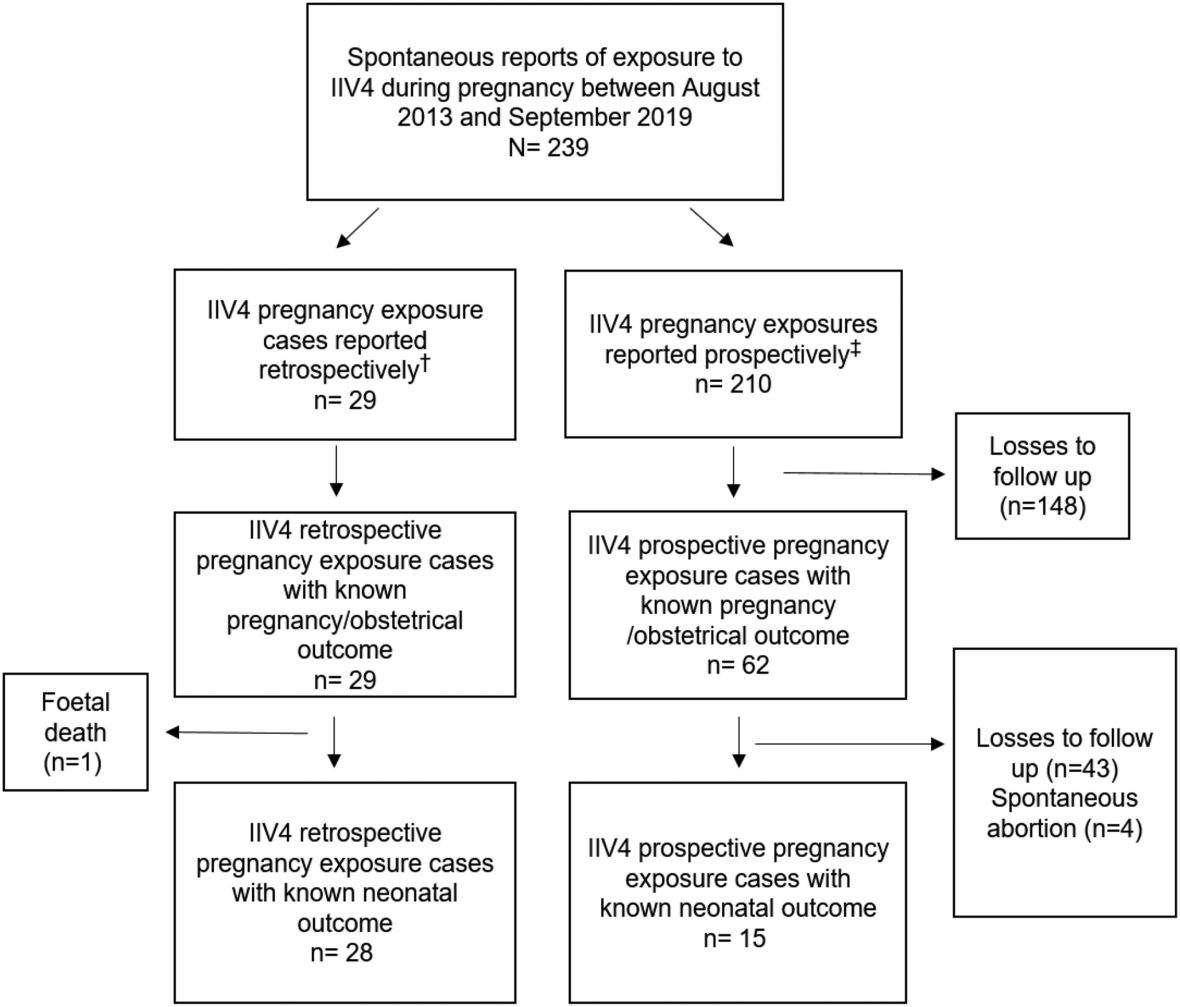
Flow diagram for reports received to the IIV4 pregnancy registry

Between August 2013 and September 2019, 239 reports of pregnancy exposure to IIV4 were captured in the registry (210 prospective and 29 retrospective reports; Figure 1). The characteristics of these spontaneous reports are summarized in Table 1. Reports were received from nine different countries, with over 85% originating from the United States, Australia and Canada; 75% of the reports were submitted by HCPs.

**TABLE 1:** Characteristics of the exposure reports (*N* = 239)

Characteristic	*n* (%)^a^
Age of pregnant women	
Mean	31.08 years
Range	16–46 years
Vaccine administered	
Fluzone QIV preservative free	227 (94.98)
Fluzone QIV preserved	12 (5.02)
Country of administration	
United States	158 (66.11)
Australia	29 (12.13)
Canada	24 (10.04)
Brazil	12 (5.02)
Mexico	10 (4.18)
New Zealand	2 (0.84)
Thailand	2 (0.84)
Costa Rica	1 (0.42)
India	1 (0.42)
Primary exposure reporter type	
Other HCP	106 (44.35)
Consumer	60 (25.10)
Physician	36 (15.06)
Pharmacist	19 (7.95)
Nurse	18 (7.53)
Gestational period at exposure	
Mean	21.52 weeks
Range	2–41 weeks
Trimester at exposure^b^	
First trimester	42 (17.57)
Second trimester	82 (34.31)
Third trimester	58 (24.27)
Unknown	57 (23.85)
Case report year	
2013	3 (1.26)
2014	13 (5.44)
2015	46 (19.25)
2016	59 (24.69)
2017	51 (21.34)
2018	47 (19.67)
2019	20 (8.37)

Abbreviations: HCP, health care practitioner; QIV, quadrivalent influenza virus.

^a^ *n* (%) unless otherwise specified.

^b^ Gestational week estimates were used to derive the trimester at exposure, where the first trimester was defined as 0–13 weeks; second trimester, 14–27 weeks; and third trimester, ≥28 weeks.

Among the 210 prospective exposure reports, 105 maternal AEs were reported for 50 pregnant women (Appendix A). The most frequent were medication errors (expired product administered: *n* = 8, 3.8%); injection site pain (*n* = 7, 3.3%); and extra dose administered (*n* = 7, 3.3%), followed by cough, fatigue, headache, pain (general) and injection site reaction (*n* = 3, 1.4% for each). Retrospective exposure reports included 10 single instances of maternal AEs (Appendix A).

There were 62 prospective reports of pregnancy and obstetrical outcomes with at least one successful follow‐up, including four spontaneous abortions (6.5%), one case of foetal hypokinesia (1.6%) and two cases of morning sickness (3.2%) (Table 2). One case of spontaneous abortion occurred in a 28‐year‐old pregnant female with a medical history of hypertension (ongoing) and anaemia, 72 days after her date of LMP and five days after vaccination. The patient was inadvertently administered an expired dose of IIV4, and a valid vaccine was administered the same day. Another case of spontaneous abortion was reported for a 31‐year‐old female with a medical history of thalassemia minor; however, relevant information such as the exact date of vaccination, the patient's date of LMP, EDD and the gestation period at the time of spontaneous abortion were missing. A 42‐year‐old woman had a spontaneous abortion at 10 weeks of gestation and 39 days after vaccination; other than advanced maternal age, the patient's medical history was unknown.

**TABLE 2:** Pregnancy and obstetrical adverse events following vaccination with IIV4

	Prospective (*N* = 62)^a^			Retrospective (*N* = 29)^b^		
	*n*	%	95% CI	*n*	%	(95% CI)
Abnormal foetal heart rate	0	—		1	3.45	0.37, 15.00^c^
Foetal death	0	—		1	3.45	0.37, 15.00^c^
Foetal hypokinesia	1	1.61	0.17, 7.29^c^	0	—	
Tachycardia foetal	0	—		1	3.45	0.37, 15.00^c^
Gestational diabetes	0	—		2	6.90	0.85, 23.03
Morning sickness	2	3.23	0.24, 11.67	0	—	
Placenta previa	0	—		1	3.45	0.37, 15.00^c^
Premature separation of the placenta	0	—		1	3.45	0.37, 15.00^c^
Spontaneous abortion	4	6.45	(2.08, 15.90)	0	—	

Abbreviation: CI, confidence interval.

^a^ Among cases with exposure reported prior to prenatal testing, with follow‐up on pregnancy/obstetrical outcomes (*N* = 62).

^b^ Among cases with exposure reported following prenatal testing, with follow‐up on pregnancy/obstetrical outcomes (*N* = 29).

^c^ Jefferies method used to ensure attained intervals fell between 0 and 1 in accordance with the binomial distribution.

A 29‐year‐old woman reported a spontaneous abortion 97 days after her LMP and 10 days after vaccination. She reported spotting four days after vaccination; pregnancy hormone levels were 28,602 eight days after vaccination and 23,608 10 days after vaccination (the day the abortion occurred). Miscarriage was reported as the outcome of pregnancy one week later. The mother's medical history and foetal pathology were not available.

Among the 29 retrospective reports of pregnancy and obstetrical outcomes with follow‐up information, seven adverse pregnancy and obstetrical AEs were described (Table 2). These included two cases (6.9%) of gestational diabetes, one case of abnormal foetal heart rate (3.4%), one case of severe premature separation of the placenta (3.4%), one case of severe placenta previa (3.4%) and one case of foetal tachycardia (3.4%). The premature separation of the placenta, placenta previa and one of the cases of gestational diabetes were experienced by a 34‐year‐old mother. At 35 weeks of gestation, 97 days after vaccination, this patient had a caesarean section and delivered a stillborn. She had a medical history of vesicular lithiasis and obesity. Postpartum follow‐up laboratory tests showed high blood glucose levels. Foetal pathology and autopsy of products of conception were not available.

Neonatal outcomes were described in 15 prospective reports and 28 retrospective reports (Table 3). A single case of premature birth was reported prospectively: a woman of unspecified age, with a history of asthma and gestational diabetes, gave birth to a baby boy at 34 weeks of gestation. The infant had a low birth weight (2.37 kg), with no congenital abnormalities, and APGAR scores of 9 at 1 minute and 10 at 5 minutes. Four AEs were reported retrospectively, including two cases of talipes (club foot), a single case of atrial septal defect and a single case of congenital central nervous system anomaly (Table 3). The first case of talipes was in a male infant born at 39 weeks of gestation and was reported after prenatal testing had occurred. The mother, aged 37 years, received IIV4 at 29 weeks of gestation. No family history of talipes and no other AEs during pregnancy were reported. The second case of talipes was reported in a male infant born to a 36‐year‐old mother who was vaccinated at six weeks of gestation. During the pregnancy, the mother had a severe respiratory infection, which was treated with amoxicillin on an unspecified date for five days. The mother's prenatal laboratory tests, prenatal vitamins and ultrasound results were not reported. The report was filed almost six months after the infant was born. The infant with a central nervous system anomaly was born to a mother of unspecified age, with no reported medical history; the exact date of vaccination was unknown. An ultrasound performed during the 17th week of pregnancy detected agenesis of the corpus callosum in the foetus.

**TABLE 3:** Neonatal Outcomes following exposure to IIV4 *in utero*

Event	Prospective^a^		Retrospective^b^	
	*n*/*M*	% (95% CI)	*n*/*M*	% (95% CI)
Congenital anomalies				
Congenital central nervous system anomaly	0/15	—	1/28	3.57 (0.39, 15.50)^c^
Atrial septal defect (patent foramen ovale)	0/15	—	1/28	3.57 (0.39, 15.50)^c^
Talipes (club foot)	0/15	—	2/28	7.14 (0.90, 23.73)
Other				
Full‐term newborn	14/15	93.33 (68.16, 100.0)	24/28	85.71 (67.89, 94.92)
Premature birth	1/15	6.67 (0.73, 27.18)^c^	0	—
Apgar score (After 5 minutes)				
Normal (≥7)	10/10	100.0 (67.91, 100.0)	22/22	100.0 (82.45, 100.0)
Low (<7)	0/10	—	0/22	—
Birth weight				
Normal (2,500–4,000 g)	7/9	77.78 (44.28, 94.66)	21/21	100.0 (81.76, 100.0)
Low (1,500–2,499 g)	2/9	22.22 (5.34, 55.72)	0/21	—
Very low (<1,500 g)	0/9	—	0/21	—

Abbreviations: CI, confidence interval; M, number of reports with information on the specified item available.

^a^ Cases with exposure reported prior to prenatal testing.

^**b**^ Cases with exposure reported following prenatal testing.

^c^ Jefferies method used to ensure attained intervals fell between 0 and 1 in accordance with the binomial distribution.

The case of atrial septal defect occurred in a female infant born at 37 weeks of gestation to a 32‐year‐old female. The mother received IIV4 199 days after her date of LMP. Ultrasound scans at eight and 20 weeks were ‘normal’. Ultrasound at 34 weeks of gestation showed irregular foetal heartbeat with premature atrial and ventricular contractions. The outcomes of central nervous system anomaly and atrial septal defect were unknown at the time of last follow‐up. Additional follow‐up information was requested. No information on the use of prenatal vitamins was available.

The APGAR score at 5 minutes was normal (≥7) for all 32 neonates with available information, and birth weight was normal (2,500–4,000 g) for 28/30 neonates. Low birth weight was reported for two infants prospectively.

Overall, 30 AEs reported for 19 pregnant women were considered an SAE (Appendix B). One SAE concerned a 31‐year‐old mother who experienced hypovolemic shock following H1N1 influenza virus infection 36 days after vaccination. The mother was vaccinated at 24 weeks of pregnancy and was considered to be a case of vaccination failure. She had a medical history of hypothyroidism treated with levothyroxine sodium. She was recovering at the time of the report, and the outcome of the pregnancy is unknown. A 29‐year‐old woman vaccinated during the third trimester of pregnancy developed Guillain–Barré syndrome (GBS) with onset five days after vaccination. She gave birth to a baby girl who was reported to be healthy and was recovering from GBS at the time of reporting. Another SAE concerned a 34‐year‐old who experienced a tonic‐clonic seizure on the day of vaccination after administration (Day 0), at 25 weeks of gestation. The patient's medical history included two previous episodes of seizures treated with medication (levetiracetam). The patient's neurologist advised an increased dosage of levetiracetam in pregnancy and reported that inadequate medication may have been the cause of seizure. The event of tonic–clonic seizure resolved on an unspecified date and pregnancy outcome was unknown. The patient's dates of LMP and EDD were not reported.

## 4 DISCUSSION

During the period from August 2013 to September 2019, 239 reports of pregnancy exposure were captured in the IIV4 pregnancy registry. Most of these were considered to be medically nonserious, and no new safety concerns were identified. Our findings are in line with listed events in the product information^28^ and previous observations of the safety of influenza vaccination during pregnancy in a number of observational studies^22^, ^23^, ^25^ and national reporting systems in the United States,^24^, ^34^ Taiwan^35^ and Australia.^36^

Among the vaccine exposure reports described here, 30 SAEs were reported for 19 women during pregnancy. These included a case of hypovolemic shock following H1N1 influenza virus infection and a case of GBS; both reported prospectively. The case of H1N1 infection reflects a vaccination failure, which would be expected to occur in a proportion of individuals after the receipt of any vaccine.^37^ Nonetheless, data for influenza seasons 2010–2016 show that influenza vaccination during pregnancy reduced the risk of being hospitalized due to influenza by 40%.^37^ GBS is a listed event for IIV4. In the case reported here, the timing of the onset of the event was compatible with the role of the vaccine. However, there is insufficient information available to determine whether this case would satisfy the criteria for the Brighton Collaboration case definition of GBS. As such, an infectious aetiology or other cause cannot be ruled out, and further assessment would be needed to confirm potential association with vaccination. Data on the association between seasonal influenza vaccination and GBS are inconsistent across seasons.^38^ During influenza seasons where an increased risk of GBS following vaccination was observed, cases were rare, ranging from one to two cases per million influenza vaccines administered.^38^

The overall frequency of occurrence of congenital abnormalities among total exposure reports described here is comparable with the estimated background of clinically recognized major birth defects in the United States general population (2%–4%).^39^ Notably, most neonatal AEs were reported retrospectively, meaning the occurrence of the event may have triggered the initial exposure report to be submitted to the registry. Among these were two cases of talipes in newborn boys. Although talipes is twice as likely to occur in male than female infants,^40^, ^41^ it occurred at a higher frequency in the current report (7.1%) than would be expected in the general population (estimated prevalence, 1 per 1,000 live births).^40^ The use of prenatal vitamins and folic acid is known to help prevent the occurrence of congenital heart defects and neural‐tube defects, including talipes.^42^ However, the use of, or absence of, prenatal vitamins was not reported for these two cases. Because one case was reported following prenatal testing and the other six months after the birth, the potential for reporting bias through retrospective reporting should be considered.

Over 65% of the pregnancy exposure reports captured here were from the United States. Therefore, the outcome frequencies can be qualitatively compared to the AEs captured in the Vaccine Adverse Event Reporting System (VAERS), a United States national vaccine passive safety surveillance system co‐managed by the United States Food and Drug Administration and the Centers for Disease Control and Prevention. Between July 2010 and May 2016, 544 reports of seasonal inactivated influenza vaccine (including 28 quadrivalent and 332 trivalent IIVs) exposure during pregnancy were reported to VAERS, which included 61 SAEs.^34^ Information on neonatal outcomes was available in 4.0% of the reports (*n* = 22), with seven reports of major birth defects (e.g., ectopic kidney, cleft lip and polydactylism, and trisomy 18).^34^ The most commonly reported pregnancy outcomes were spontaneous abortion (11.4%), stillbirth (1.8%) and preterm delivery (1.1%). Similarly, in a national passive surveillance system for AEs following immunization established during the 2009 H1N1 vaccination programme in Taiwan,^35^ 16 spontaneous abortions, 11 stillbirths and four neonatal deaths were reported between 2009 and 2010. Authors estimated the risk of spontaneous abortion was 2.3/100 pregnancies, compared with a local background rate of 12.8/100 pregnancies.^35^ Notably, the rate of spontaneous abortion among pregnant women described in the current registry is no higher than background rates of miscarriage in clinically recognized pregnancy in the United States general population (15%–20%).^39^

The IIV4 pregnancy registry is a passive pharmacovigilance surveillance system; as such, our data are subject to the limitations of passive surveillance that have been previously well‐described.^43^, ^44^ These are likely to have resulted in an underreporting of pregnancy exposures overall in this analysis. However, among the reports submitted, there is a greater likelihood that serious events, such as foetal death and talipes, and events occurring closer to time of vaccination were reported. Women who experienced one or more SAE are also more likely to have reported to the pregnancy registry, meaning that overreporting of SAEs would be expected among the retrospective reports. A high rate of loss to follow‐up (approximately 80% in the current study), due to a variety of factors such as incorrect or missing contact information, the submission of reports from pharmacists without access to personal information and unwillingness to participate, may additionally affect interpretation of these findings.

## 5 CONCLUSION

The frequency of maternal, pregnancy, obstetrical and neonatal adverse outcomes following exposure to IIV4 during pregnancy are consistent with those reported elsewhere and do not exceed the expected rates in the general population. There were no new safety concerns identified, supporting the established positive benefit‐risk profile of IIV4.

## AUTHOR CONTRIBUTIONS

**Shaleesa Ledlie:** Data curation; formal analysis; investigation; methodology; writing‐review and editing. **Sonja Gandhi‐Banga:** Conceptualization; investigation; methodology; writing‐review and editing. **Anju Shrestha:** Formal analysis; investigation; writing‐review and editing. **Tamala Mallett Moore:** Formal analysis; investigation; writing‐review and editing. **Alena Khromava:** Conceptualization; investigation; methodology; writing‐review and editing.

## ETHICS APPROVAL STATEMENT/PATIENT CONSENT STATEMENT

This study represents routine pharmacovigilance, and as such was not subject to Institutional Review Board review and informed consent requirements.

### PEER REVIEW

The peer review history for this article is available at https://publons.com/publon/10.1111/irv.12897.

## Data Availability

Qualified researchers may request access to patient‐level data and related study documents including the clinical study report, study protocol with any amendments, blank case report form, statistical analysis plan and dataset specifications. Patient‐level data will be anonymized, and study documents will be redacted to protect the privacy of trial participants. Further details on Sanofi's data sharing criteria, eligible studies and process for requesting access can be found online: https://www.clinicalstudydatarequest.com.

## APPENDIX A MATERNAL ADVERSE EVENTS FOLLOWING VACCINATION WITH IIV4

**Table:**

	Prospective (*N* = 210)^a^			Retrospective (*N* = 29)^b^		
	*n*	%	95% CI	*n*	%	95% CI
Asthenia	1	0.48	0.05, 2.20^c^	—	—	—
Chest discomfort	1	0.48	0.05, 2.20^c^	—	—	—
Cough	3	1.43	0.29, 4.31	—	—	—
Dizziness	2	0.95	0.04, 3.63	—	—	—
Dry Throat	1	0.48	0.05, 2.20^c^	—	—	—
Dyspnoea	2	0.95	0.04, 3.63	—	—	—
Expired product administered	8	3.81	1.82, 7.46	1	3.45	0.37, 15.00^c^
Extra dose administered	7	3.33	1.49, 6.85	1	3.45	0.37, 15.00^c^
Eye pruritus	1	0.48	0.05, 2.20^c^	—	—	—
Facial pain	1	0.48	0.05, 2.20^c^	—	—	—
Fatigue	3	1.43	0.29, 4.31	—	—	—
Fibromyalgia	1	0.48	0.05, 2.20^c^	—	—	—
Pain (general)	3	1.43	0.29, 4.31	—	—	—
Gingival pain	1	0.48	0.05, 2.20^c^	—	—	—
Guillain–Barré syndrome	1	0.48	0.05, 2.20^c^	—	—	—
H1N1 influenza infection	2	0.95	0.04, 3.63	—	—	—
Headache	3	1.43	0.29, 4.31	—	—	—
Hypersensitivity	1	0.48	0.05, 2.20^c^	—	—	—
Hypaesthesia	1	0.48	0.05, 2.20^c^	—	—	—
Hypoglycaemia	1	0.48	0.05, 2.20^c^	1	3.45	0.37, 15.00^c^
Inappropriate concomitant administration of additional vaccine	2	0.95	0.04, 3.63	—	—	—
Injection site inflammation	3	1.43	0.29, 4.31	—	—	—
Influenza infection	2	0.95	0.04, 3.63	1	3.45	0.37, 15.00^c^
Influenza‐like illness	2	0.95	0.04, 3.63	—	—	—
Injection site bruising	1	0.48	0.05, 2.20^c^	—	—	—
Injection site mass	1	0.48	0.05, 2.20^c^	—	—	—
Injection site pain	7	3.34	1.17, 6.24	1	3.45	0.37, 15.00^c^
Injection site reaction	3	1.43	0.29, 4.31	—	—	—
Injection site scar	1	0.48	0.05, 2.20^c^	—	—	—
Lupus enteritis	1	0.48	0.05, 2.20^c^	—	—	—
Malaise	2	0.95	0.04, 3.63	—	—	—
Myalgia	1	0.48	0.05, 2.20^c^	—	—	—
Nasal congestion	1	0.48	0.05, 2.20^c^	—	—	—
Nasopharyngitis	1	0.48	0.05, 2.20^c^	1	3.45	0.37, 15.00^c^
Pain in extremities	1	0.48	0.05, 2.20^c^	—	—	—
Presyncope	1	0.48	0.05, 2.20^c^	—	—	—
Product storage error	3	1.43	0.29, 4.31	1	3.45	0.37, 15.00^c^
Pruritus	2	0.95	0.04, 3.63	—	—	—
Pyrexia	2	0.95	0.04, 3.63	1	3.45	0.37, 15.00^c^
Rash	2	0.95	0.04, 3.63	—	—	—
Rash maculopapular	1	0.48	0.05, 2.20^c^	—	—	—
Rash papular	2	0.95	0.04, 3.63	—	—	—
Rash pruritic	1	0.48	0.05, 2.20^c^	—	—	—
Rhinorrhoea	2	0.95	0.04, 3.63	—	—	—
Secondary infection	1	0.48	0.05, 2.20^c^	—	—	—
Seizure	1	0.48	0.05, 2.20^c^	—	—	—
Shock	1	0.48	0.05, 2.20^c^	—	—	—
Sinusitis	2	0.95	0.04, 3.63	—	—	—
Skin discoloration	2	0.95	0.04, 3.63	—	—	—
Sneezing	1	0.48	0.05, 2.20^c^	—	—	—
Thrombocytopenia	1	0.48	0.05, 2.20^c^	—	—	—
Underdose	1	0.48	0.05, 2.20^c^	—	—	—
Upper respiratory tract infection	—	—	—	1	3.45	0.37, 15.00^c^
Urticaria	1	0.48	0.05, 2.20^c^	—	—	—
Vaccination failure	1	0.48	0.05, 2.20^c^	—	—	—
Vaccination site erythema	2	0.95	0.04, 3.63	—	—	—
Vaccination site Inflammation	2	0.95	0.04, 3.63	—	—	—
Vaccination site pruritus	1	0.48	0.05, 2.20^c^	—	—	—
Vaccination site swelling	—	—	—	1	3.45	0.37, 15.00^c^
Vomiting	1	0.48	0.05, 2.20^c^	—	—	—
Wheezing	1	0.48	0.05, 2.20^c^	—	—	—

^a^ Among cases with exposure reported prior to prenatal testing (*N* = 210).

^b^ Among cases with exposure reported following prenatal testing (*N* = 29).

^c^ Jefferies method used to ensure attained intervals fell between 0,1 in accordance with the binomial distribution.

## APPENDIX B MEDICALLY SERIOUS ADVERSE EVENTS FOLLOWING VACCINATION WITH QUADRIVALENT INFLUENZA VIRUS (QIV)

**Table:**

Case number	Outcome	Time to onset, days	Outcome^a^
2013SA100435	Wheezing	0	Recovered
	Chest Discomfort	0	Recovered
	Dyspnoea	0	Recovered
	Dry Throat	0	Recovered
	Cough	0	Recovered
2013SA117044	Gestational diabetes	Unknown	Unknown
2018SA164289	Shock	Unknown	Recovered
	H1N1 influenza	36	Recovered
	Vaccination failure	36	Not applicable
	Thrombocytopenia	Unknown	Recovered
2018SA258415	Abortion spontaneous	Unknown	Unknown
2018SA280579	Seizure	0	Recovered
2019SA011506	Fibromyalgia	Unknown	Unknown
2019SA060034	Influenza infection	Unknown	Unknown
2019SA128778	Rash Maculopapular	1	Recovered
2019SA248317	Abortion spontaneous	Unknown	Unknown
2018SA186971	Congenital central nervous system anomaly	Unknown	Unknown
2015SA177203	Talipes	Unknown	Unknown
2015SA047942	Atrial septal defect (patent foramen ovale)	Unknown	Unknown
2016SA085413	H1N1 influenza	6	Recovered
2016SA099610	Guillain–Barré syndrome	5	Recovered
2017SA012845	Abortion spontaneous	39	Unknown
2017SA032874	Foetal death	97	Not applicable
	Gestational diabetes	Unknown	Unknown
	Placenta previa	Unknown	Recovered
	Premature abruption	Unknown	Recovered
2017SA032833	Talipes	228	Recovered
	Upper respiratory tract infection	Unknown	Recovered
2017SA237500	Foetal hypokinesia	0	Unknown
2019SA035906	Spontaneous abortion	10	Recovered

^a^ As per last follow‐up.
